# Clinical Holistic Medicine (Mindful, Short-Term Psychodynamic Psychotherapy Complemented with Bodywork) in the Treatment of Experienced Physical Illness and Chronic Pain

**DOI:** 10.1100/tsw.2007.68

**Published:** 2007-03-02

**Authors:** Søren Ventegodt, Suzette Thegler, Tove Andreasen, Flemming Struve, Lars Enevoldsen, Laila Bassaine, Margrethe Torp, Joav Merrick

**Affiliations:** ^1^ Quality of Life Research Center, Teglgårdstræde 4-8, DK-1452 Copenhagen K, Denmark; ^2^ Research Clinic for Holistic Medicine, Copenhagen, Denmark; ^3^ Nordic School of Holistic Medicine, Copenhagen, Denmark; ^4^ Scandinavian Foundation for Holistic Medicine, Sandvika, Norway; ^5^ Interuniversity College, Graz, Austria; ^6^ Zusman Child Development Center, Soroka University Medical Center, Ben Gurion University of the Negev, Beer-Sheva, Israel; ^7^ National Institute of Child Health and Human Development, Jerusalem, Israel; ^8^ Office of the Medical Director, Division for Mental Retardation, Ministry of Social Affairs, Jerusalem, Israel

**Keywords:** pain, quality of Life, CAM, psychodynamic therapy, bodywork, Denmark, shortterm psychodynamic psychotherapy (STPP), holistic medicine, existential healing, salutogenesis, Antonovsky

## Abstract

We investigated the treatment effect of psychodynamic short-term therapy complemented with bodywork on patients who presented with physical illness at the Research Clinic for Holistic Medicine in Copenhagen. Psychodynamic short-term therapy was complemented with bodywork (Marion Rosen) to help patients confront old emotional pain from childhood trauma(s). Patients were measured with a five-item quality of life and health questionnaire (QOL5), a one-item questionnaire of self-assessed quality of life (QOL1), and four questions on self-rated ability to love and to function sexually, socially, and at work (ability to sustain a full-time job). Most of the patients had chronic pain that could not be alleviated with drugs. Results showed that 31 patients with the experience of being severely physically ill (mostly from chronic pain), in spite of having consulted their own general practitioner, entered the study. The holistic approach and body therapy accelerated the therapy dramatically and no significant side effects were detected. After the intervention, 38.7% did not feel ill (1.73 < NNT < 4.58) (p = 0.05). Psychodynamic short-term therapy complemented with bodywork can help patients. When the patients responded to the therapy, the self-assessed mental health, relationship with partner, ability to work, self-assessed quality of life, relationships in general, measured QOL (with the validated questionnaire QOL5), and life's total state (mean of health, QOL and ability) were significantly improved, statistically and clinically. Most importantly, all aspects of life were improved simultaneously, due to induction of Antonovsky-salutogenesis. The patients received in average 20 sessions over 14 months at a cost of 1600 EURO. For the treatment responders, the treatment seemingly provided lasting benefits.

## INTRODUCTION

The famous physician Hippocrates (460–377 BCE) cured his patients using holistic medicine, where he emphasized addressing the whole person. His primary tool, according to the Corpus Hippocraticum[1], was rehabilitation of the character. His medicine became the medicine of the west for more than 2 millenniums. Since 1950, biomedicine (using drugs to cure) has become more popular than holistic medicine, but patients unsatisfied with the results and the side effects of biomedicine have recently returned to the "old way" of being healed.

Since 1997, we have conducted clinical research in holistic medicine to develop the psychodynamic short-term therapy known from several meta-studies to help many different groups of patients[2,3,4,5,6,7]. We have developed this therapy into a fast and efficient holistic tool for the general practitioner to be able to help patients with physical, mental, existential, and sexual problems[8,9,10,11,12]. We want a medicine that is fast, efficient, affordable, preventive, safe, and gives lasting results and, if possible, we would also like to improve not only health, but also the quality of life and general ability of functioning, especially the ability to work happily. This is much to ask from a medicine, but according to Aaron Antonovsky's (1923–1994) theory of *"salutogenesis"*[13,14] — or *healing of the patient's whole life* — this is actually possible. In 2000, we established the Research Clinic for Holistic Medicine in Copenhagen to develop and document a clinical holistic medicine–inducing salutogenesis with now more than 500 patients treated in a 10-year clinical study planned to include 1,000 patients. To simplify, we decided to use the patients' self-evaluated health as both a diagnostic and documentary tool, as many studies indicated self-assessed health to be one of the strongest indications for present and future health, if not the strongest[15,16,17,18,19].

Most often, the patients in our clinic have a complex of mental, physical, existential, and sexual problems that have often become chronic and disabling problems. The patients came to us after reading our books on holistic medicine[8,9,10] or through recommendations from other patients. They have to pay for the treatment (the clinic is not part of the national health system), where, on average, 20 sessions cost about 1,600 EURO.

## METHODS

Since the year 2000, we have treated more than 500 patients with "clinical holistic medicine". In the year 2004–2005, we treated 109 patients and, among these, 31 had significant physical health issues when measured on a 5-point Likert scale (1 as "very good" to 5 as "very bad") for self-rated physical health. The patients were measured with a five-item quality of life and health questionnaire QOL5 (five questions on self-assessed physical health, mental health, relation to self, relation to partner, and relation to friends[20]), a one-item questionnaire of self-assessed quality of life (QOL1)[20], and four questions on self-rated ability to love and to function sexually, socially, and at work (ability to sustain a full-time job) (together, QOL10 questionnaire).

The patients were questioned before entering the study, after the treatment, and after 1 year[21,22]. The patients who had rated themselves to be physically ill and entered the clinic in 2004–2005 were part of this present study. Six therapists from the Nordic School of Holistic Medicine (master students)

performed the therapy as research associates under supervision by the first author. Many more of the 109 patients than the 31 patients selected for this study had physical health issues — chronic pain in the locomotor system (26), chronic pain in the internal organs (25), asthma (4), allergy (9), eczema (5), chronic infections (7), autoimmune diseases (4), genetic disorders (2), female hormonal disturbances (bleeding) (5) — but we only included the patients who rated themselves as "physically ill". Some of these patients had more than one disease, and some patients who felt severely ill were still without a diagnosis in spite of a thorough examination by their own general practitioner. The patients most often felt ill from chronic pains, without any specific location in the body (wandering pain, nonanatomical pain). The patients included in this study were the difficult-to-treat, chronic patients that are all too well known in general practice and who almost never improve spontaneously, nor respond well to drugs.

## RESULTS

Table 1 shows that 31 patients were physically ill before treatment (self-assessed physical health: bad or very bad), 12 patients were physically well after treatment (self-assessed physical health: very good, good, or neither good nor bad), and six patients were still physically ill after treatment (self-assessed physical health: bad or very bad), while 13 did not respond to the follow-up questionnaire. Response rate of follow-up study was, therefore, 58.1%.

**TABLE 1**
**Clinical Holistic Medicine Cure 38.71% ($p$ = 0.05 or 95% CI: 22–58%) of Patients from Self-Assessed Somatic Illness**

| | Before Treatment | After Treatment |
|---|---|---|
| Physically ill | 31 | 6 |
| Physically well | 0 | 12 = 38.71%; 95% CI: 22–58% |
| Nonresponders or dropouts | — | 13 |

Success rate of treatment: 12/31 = 38.71% (95% CI: 21.85–57.81%)[23]. Calculated in this way, we get an NNT (Number Needed to Treat) of clinical holistic medicine with somatically ill patients = 1.73–4.58. As we have treated over 500 patients with no severe or lasting side effects (see below) and no patients harmed, we estimate the Number Needed to Harm (NNH) to be >500 (we do not have the follow-up data on these patients).

The self-assessed mental health, relationship with partner, ability to work, self-assessed quality of life (see Tables 2 and 3), relationships in general, measured QOL (with the validated questionnaire QOL5), and total state of life (mean of health, QOL, and ability) were also significantly improved, statistically and clinically (see Tables 4 and 5).

When we used paired T-test to study the 12 patients, where therapy changed their ratings of self-evaluated physical health from ill to not ill (from 4 = bad or 5 = very bad, to 1 = very good, 2 = good, or 3 = neither good nor bad), we found (see Tables 2 and 3) that the 12 patients also improved their self-evaluated mental health, relationship with partner, working ability, and quality of life. Please note that the results are both statistically and clinically highly significant. In Tables 4 and 5, it can be seen that they also improved their relations (with self, partner, and friends), their self-evaluated ability to function (to love, to function sexually, socially, and at work), and their quality of life as measured with the validated questionnaire QOL5. When health, quality of life, and ability were combined, it became clear that these patients had healed their whole life. This healing of all aspects of life is often seen in clinical holistic medicine and called Antonovsky-salutogenesis, after the researcher who discovered this kind of global healing of the patient's existence[13,14].

**TABLE 2**
**Study of 12 Patients where Therapy Changed their Ratings of Self-Evaluated Physical Health from Ill to Not Ill (Paired Samples)**

| | | Mean | N | Std. | Std. Mean |
|---|---|---|---|---|---|
| Physical health | Before | 4.000 | 12 | 0.0000 | 0.0000 |
| | After | 2.666 | 12 | 0.4923 | 0.1421 |
| Mental health | Before | 3.500 | 12 | 0.6742 | 0.1946 |
| | After | 2.250 | 12 | 0.7537 | 0.2176 |
| Self-esteem | Before | 3.000 | 12 | 0.8528 | 0.2461 |
| | After | 2.500 | 12 | 0.5222 | 0.1507 |
| Relation to friends | Before | 2.416 | 12 | 0.9003 | 0.2599 |
| | After | 2.250 | 12 | 0.4522 | 0.1305 |
| Relation to partner | Before | 3.666 | 12 | 1.8748 | 0.5412 |
| | After | 2.666 | 12 | 1.7232 | 0.4974 |
| Ability to love | Before | 3.250 | 12 | 0.9653 | 0.1786 |
| | After | 2.583 | 12 | 0.5149 | 0.1486 |
| Sexual ability | Before | 3.500 | 12 | 1.0871 | 0.3138 |
| | After | 2.833 | 12 | 0.9374 | 0.2706 |
| Social ability | Before | 3.166 | 12 | 0.9374 | 0.2706 |
| | After | 2.333 | 12 | 0.7785 | 0.2247 |
| Work ability | Before | 3.166 | 12 | 0.8348 | 0.2410 |
| | After | 2.333 | 12 | 0.6513 | 0.1880 |
| Quality of life | Before | 3.916 | 12 | 0.5149 | 0.1486 |
| | After | 2.500 | 12 | 0.7977 | 0.2302 |

## DISCUSSION

In an earlier paper, we documented that the treatment effect is lasting, using the square curve paradigm; the results do not deteriorate 1 year after therapy[21]. After the treatment, we found that 38.7% did not feel ill any more, if we calculate our success rate conservatively, and take all dropouts and nonresponders of the follow up questionnaire as negative responders to the treatment.

Of the responders, 33.3% felt completely cured (good or very good) and 66.7% was improving (felt neither bad nor good). Most importantly, all aspects of life improved simultaneously because of induction of Antonovsky-salutogenesis[13,14]. The treatment of these patients with clinical holistic medicine had no side effects except for a few days of feeling bad when old painful, repressed material from old trauma reappeared in the consciousness of the patient. During the most intense phase of therapy, many patients felt very bad for a few days, but none of the patients experienced severe or lasting side effects. Two patients needed support 24 h/day for a few days during this period.

The problem of being ill has three major aspects: the symptoms, the compromised quality of life, and the reduced ability to function. Another important aspect is that illness often signifies a continuous deterioration of life through time. Disease or being ill invites new illness and loss of quality of life and ability.

**TABLE 3**
**Paired Samples**

| | Paired Differences | | | | | t | df | Significance (Two–Tailed) |
|---|---|---|---|---|---|---|---|---|
| | Mean | Std. Deviation | Std. Error Mean | 95% CI of Difference | | | | |
| | | | | Lower | Upper | | | |
| Physical health | 1.3333 | 0.49237 | 0.14213 | 1.0205 | 1.6462 | 9.381 | 11 | 0.000 |
| Mental health | 1.2500 | 1.05529 | 0.30464 | 0.5795 | 1.9205 | 4.103 | 11 | 0.002 |
| Self-esteem | 0.5000 | 1.08711 | 0.31382 | –0.1907 | 1.1907 | 1.593 | 11 | NS |
| Relation to friends | 0.1667 | 0.57735 | 0.16667 | –0.2002 | 0.5335 | 1.000 | 11 | NS |
| Relation to partner | 1.0000 | 1.47710 | 0.42640 | 0.0615 | 1.9385 | 2.345 | 11 | 0.039 |
| Ability to love | 0.6667 | 1.15470 | 0.33333 | –0.0670 | 1.4003 | 2.000 | 11 | NS |
| Sexual ability | 0.6667 | 1.4570 | 0.33333 | –0.0670 | 1.4003 | 2.000 | 11 | NS |
| Social ability | 0.8333 | 1.33712 | 0.38599 | –0.0162 | 1.6829 | 2.159 | 11 | NS |
| Work ability | 0.8333 | 0.71774 | 0.20719 | 0.3773 | 1.2894 | 4.022 | 11 | 0.002 |
| Quality of life | 1.4167 | 0.99620 | 0.28758 | 0.7837 | 2.0496 | 4.926 | 11 | 0.000 |

**TABLE 4**
**Study of 12 Patients where Therapy Changed their Ratings of Self-Evaluated Physical Health from Ill to Not Ill (Paired T-Test)**

| | | Mean | N | Std. | Std. Mean |
|---|---|---|---|---|---|
| Relations | Before | 3.027 | 12 | 0.7029 | 0.2029 |
| | After | 2.472 | 12 | 0.6106 | 0.1762 |
| Ability | Before | 3.270 | 12 | 0.6436 | 0.1858 |
| | After | 2.520 | 12 | 0.4579 | 0.1322 |
| QOL (QOL 5) | Before | 3.263 | 12 | 0.5097 | 0.1471 |
| | After | 2.472 | 12 | 0.4428 | 0.1278 |
| Health-QOL-Ability (QOL 10) | Before | 3.349 | 12 | 0.4804 | 0.1387 |
| | After | 2.483 | 12 | 0.4027 | 0.1162 |

The most fundamental problem with holistic medicine is that it addresses the patient’s consciousness and experience (the whole person), and not the objective symptoms and diseases. When a person is cured in this system, the patient feels cured. The literature documents a strong correlation between self-assessed and objective health. The danger here is that the patient might abandon an important biomedical treatment, and it is therefore mandatory that clinical holistic medicine should be seen as a supplement to biomedicine and performed with physician supervision. To induce holistic healing, or salutogenesis[13,14], the most fundamental beliefs of the patient (the patient’s philosophy of life) must be addressed and changed toward a more positive and life-sustaining one. To do this, the repressed feelings and emotions of the trauma(s) that established the existential damage to begin with must be integrated. The process is often very dramatic and often emotionally painful for the patient, and the patient needs to work hard with exercises that are most often writings and readings. The reward from this suffering seems to be a general improvement, not only of physical health, but also of all other aspect of life. The process of existential healing is arduous and painful, which limits the number of patients who are willing to use this kind of medicine/treatment or indeed induced self-treatment.

**TABLE 5**
**Paired Samples**

| | Paired Differences | | | | | t | df | Significance (Two–Tailed) |
|---|---|---|---|---|---|---|---|---|
| | Mean | Std. Deviation | Std. Error Mean | 95% CI of Difference | | | | |
| | | | | Lower | Upper | | | |
| Relations | 0.5556 | 0.70113 | 0.20240 | 0.1101 | 1.0010 | 2.745 | 11 | 0.019 |
| Ability | 0.7500 | 0.72300 | 0.20871 | 0.2906 | 1.2094 | 3.593 | 11 | 0.004 |
| QOL (QOL 5) | 0.7917 | 0.58657 | 0.16933 | 0.4190 | 1.1644 | 4.675 | 11 | 0.001 |
| Health-QOL-Ability (QOL 10) | 0.8657 | 0.56925 | 0.16433 | 0.5041 | 1.2274 | 5.268 | 11 | 0.000 |

## CONCLUSIONS

Clinical holistic medicine is especially useful with chronic patients that present the triad of low quality of life, poor health (physical and/or mental), and poor ability to function. We found that patients who experienced being physically ill, in spite of having consulted their own general practitioner, entered this study and after holistic treatment, 38.7% did not feel ill any longer ($1.73 < NNT < 4.58$) ($p = 0.05$). From more than 500 patients treated with no severe side effects, we estimate that NNH > 500. The self-assessed mental health, relationship with partner, ability to work, self-assessed quality of life, relationships in general, measured QOL (with the validated questionnaire QOL5), and life's total state (mean of health, QOL and ability) were significantly improved, statistically and clinically. These results are comparable to the results from established treatments. Most importantly, all aspects of life are improved simultaneously because of induction of Antonovsky-salutogenesis. Clinical holistic medicine has no known side effects, it seems to give fast and lasting results, and might prevent future disease.

## ACKNOWLEDGMENTS

This study was supported by grants from IMK Almene Fond. The quality of life research was originally approved by the Copenhagen Scientific Ethical Committee under number (KF)V.100.2123/91 and later correspondence.
